# Integrating molecular biomarkers and ectoparasitic infestation to assess water quality

**DOI:** 10.1038/s41598-025-20433-8

**Published:** 2025-10-07

**Authors:** Hamada S. Salem, Rana H. Omar, Ahmed M. El-Naggar, Amira A. Ibrahim, Mohamed I. Mashaly

**Affiliations:** 1 Lecturer of Environmental Science, Department of Zoology, Faculty of Science, Mansoura University, Egypt, 35516 Egypt; 2 Assistant Lecturer of Environmental Science, Department of Zoology, Faculty of Science, Mansoura University, Mansoura, 35516 Egypt; 3 Professor of Environmental Science, Department of Zoology, Faculty of Science, Mansoura University, Mansoura, 35516 Egypt; 4 Lecturer of Molecular Biology, Botany and Microbiology Department, Faculty of Science, El-Arish University, El-Arish, 45512 Egypt; 5 Associate Professor of Parasitology, Department of Zoology, Faculty of Science, Mansoura University, Mansoura, 35516 Egypt

**Keywords:** Bioindicators, Ectoparasitic infestation, Water quality assessment, Oxidative stress, Inflammation biomarkers, Ecophysiology, Freshwater ecology, Molecular ecology

## Abstract

Fish and their ectoparasites serve as model organisms for evaluating aquatic ecosystem health and can thus function as bioindicators of environmental pollution. This study, therefore, investigates the gene expression of biomarkers of effect in *Clarias gariepinus* inhabiting a polluted drain called the Kitchener Drain and the Nile River (a reference habitat). Ectoparasites and metacercariae of digenean endoparasites on the collected fish were examined to further clarify the impact of pollution. Compared with the Nile River, the Kitchener Drain contained significantly elevated concentrations of heavy metals, bicarbonates, sulfates, and minerals. Interestingly, the prevalence, mean intensity, and abundance of ectoparasitic monogeneans from *C. gariepinus* collected from the Nile River were higher compared with those from the Kitchener Drain, whereas the drain had higher levels of metacercaria of the digenean endoparasite *Centrocestus formosanus*. Fish from the Kitchener Drain had significantly elevated hepatic expressions of genes related to metal toxicity, oxidative stress, inflammation, heat shock response, and xenobiotic metabolism. The results indicate that *C. gariepinus* exposed to metals and other pollutants triggers an adaptive response involving multiple stress-response pathways. The findings also highlight the impact of pollution on parasitic infestation in this fish population. The study supports the utilization of molecular biomarkers and ectoparasitic infestation as early warning signals for water pollution.

## Introduction

Over recent decades, industrial activities have increased dramatically, especially in developing countries, to cope with the requirements of a growing population worldwide^1^. The increased industrial activity caused a dramatic increase in the discharge of industrial solid waste and household waste to aquatic ecosystems^1^. Such wastewaters contain heavy metals, organic pollutants, and other contaminants, and are extremely hard to treat, demanding expensive advanced technologies and a lot of energy inputs^1,2^. Therefore, vast quantities of untreated or poorly treated wastewater tend to be discharged into streams and drainage^2^.

Long-term exposure to water pollutants and stressors negatively impacts fish, impairing their metabolic functions and decreasing growth, performance, and survivability^3^. To survive in contaminated environments, fish undergo biochemical and physiological changes to ensure homeostasis and protect themselves against the effects of pollutants^4^. Their sensitivity to water contamination makes fish valuable bioindicators for assessing pollution levels^4,5^. Chemical stress exposure can be detected by molecular biomarkers in different species of fish^5^.

Furthermore, variability in water and environmental conditions causes varying degrees of parasitic infestations in fish, rendering the parasites and the fish effective biological indicators of the health of the environment^6^. Monogenean parasites are especially useful indicators of the quality of the environment because their numerical responses are predictable against chemical pollution^7,8^. They are resistant to low-to-moderate concentrations of pollutants but disappear with increasing concentrations^7,8^.

In this study, *Clarias gariepinus* (*C. gariepinus*) was employed as a bioindicator to assess water pollution caused by heavy metals and other contaminants. Also, ectoparasitic monogeneans as well as metacercariae of digenean endoparasites on the collected fish were examined to further determine the impact of pollution. The expression of various genetic biomarkers in the liver of *C. gariepinus* was examined to assess the multifaceted impact of pollutants on the adaptive responses of fish.

## Materials and methods

### Study area

**Fig. 1:**
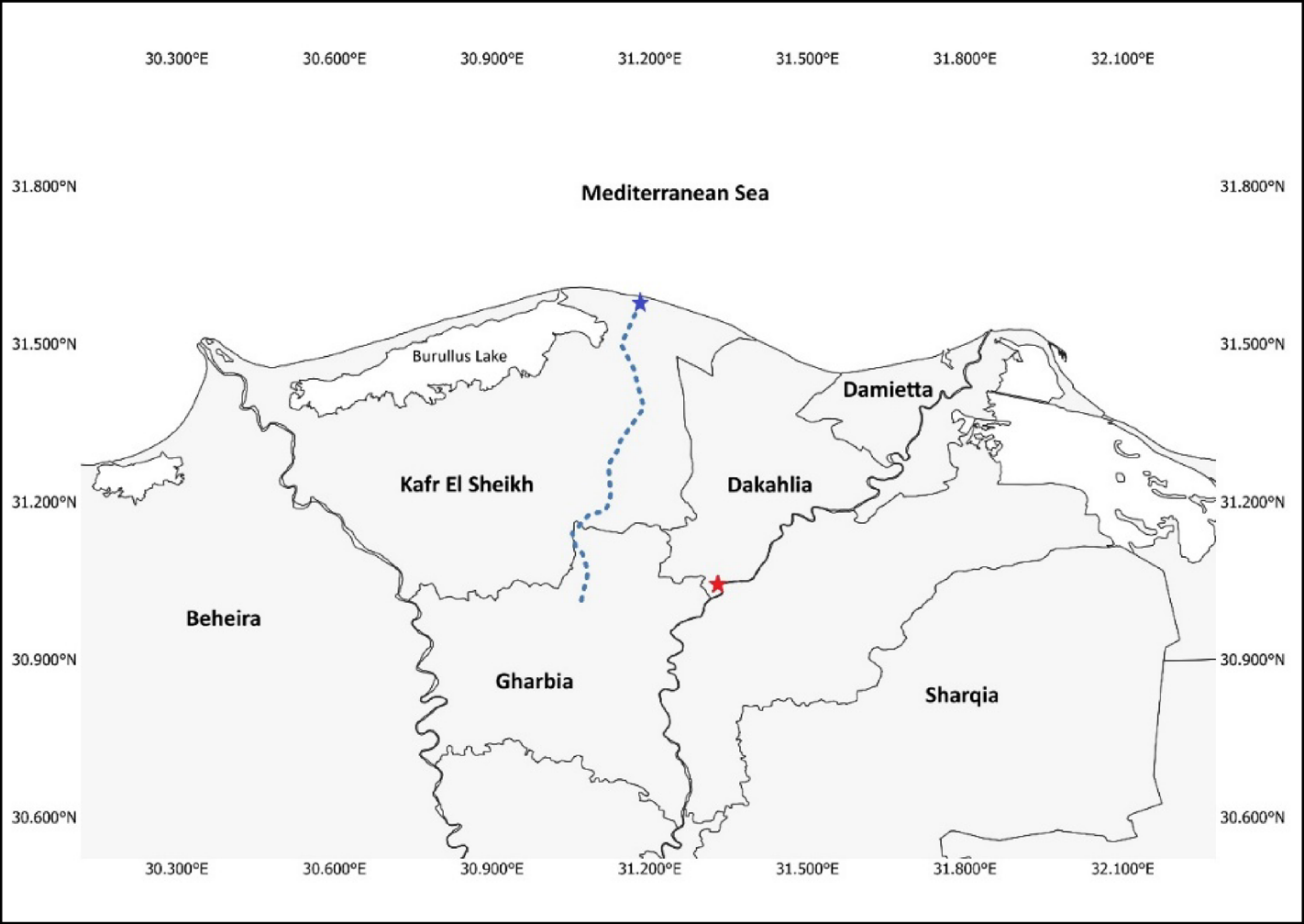
The study area included two aquatic habitats: The Damietta branch of the River Nile at Meet Badr Khamis, Dakahlia Governorate (Red Star) and the Kitchener Drain (the dashed line), Kafr El Sheikh Governorate (Blue Star). The map was generated using QGIS (version 3.44.2). The administrative boundary data was sourced from the United Nations Office for the Coordination of Humanitarian Affairs via the Humanitarian Data Exchange and is available under a CC BY-IGO license (https://data.humdata.org/dataset/cod-ab-egy.

The study area included two aquatic habitats. The first habitat was the Damietta branch of the River Nile at Meet Badr Khamis, Dakahlia Governorate (31°02’44.8"N 31°20’58.1"E). The second polluted habitat was the Kitchener Drain (downstream), Kafr El Sheikh Governorate (31°34’31.7"N 31°10’57.8"E) (Fig. 1), where the drain joins the Mediterranean coast. Sampling and analysis procedures were performed between Autumn 2021 and Summer 2022.

### Water sampling

Subsurface water samples were collected seasonally (twice per season) from each site in polyethylene bottles at a depth of 25 cm. Each sample was split into two portions: one Liter was stored at 4 °C in a polyethylene bottle for subsequent physicochemical analysis, while approximately 100 mL was stabilized with 3 mL of nitric acid (HNO_3_) and stored in a glass bottle for later heavy metal analysis.

### Sampling of the host fish

This study was approved by and performed following the guidelines provided by the Mansoura University Animal Care and Use Committee (Approval No. Sci-Z-M-2021-73). The study is also reported in accordance with ARRIVE guidelines. Between Autumn 2021 and Summer 2022, 198 specimens of *C. gariepinus* Burchell, 1822 were gathered from the two sites using fishing nets. Alive fish specimens were transported to the laboratory for Liver sample extraction for RT-PCR analysis. The remaining specimens were preserved immediately in 10% formalin and taken to the laboratory for examination of monogenean parasites and digenean metacercariae.

### Physicochemical analysis

Water temperature, electrical conductivity (EC), hydrogen ion concentration (pH), dissolved oxygen (DO), turbidity, and depth were assessed. Turbidity was assessed in situ by a Secchi disk. Hydrogen ion concentration and temperature were measured in situ using a waterproof temperature/pH meter (HI991001, Hanna). DO was measured by a Lutron YK-22DOA DO meter. A multi-parameter analyzer (Model C535, Consort) was utilized to assess EC. A PFP7 flame photometer (Jenway) was employed to determine the following elements: calcium, sodium, and potassium^9^. Chlorides, sulfates, bicarbonates, magnesium, phosphorus, and nitrogen were estimated according to the American Public Health Association^10^. Blanks and certified standards were used to check for accuracy.

### Heavy metals analysis

According to Nasrabadi et al.^11^, the concentrations of Al, V, Cr, Cu, Ga, Fe, Ag, Hg, B, Cd, Ba, Co, In, Li, Sr, Zn, As, Mn, Ni, Pb, Bi, and Se were determined utilizing inductively coupled plasma optical emission spectrometry (ICP-OES; iCAP™ 7000 Plus Series, Thermo Scientific™) after digestion with 30% HCl and 69% HNO_3_ in a microwave digestion unit (MLS 1200 Mega, Milestone). Calibration of instruments and assurance of measurement precision and accuracy were achieved using appropriate blanks and standard solutions. The selection of wavelengths for ICP-OES analysis was guided by ISO 11,885^12^. All glassware was soaked overnight in 10% HNO_3_, rinsed with deionized water, and air-dried before use^13^. All chemicals employed were of analytical reagent grade.

### Water quality index

The water quality for surface water was evaluated in the Nile and the drain using the Canadian Council of Ministers of the Environment (CCME) water quality index (WQI)^14^. This index was selected for its ability to convert water-quality data across different measurement units into a simple rating scale that reflects the condition of water (e.g., Excellent, Good, Fair, Marginal, Poor)^14,15^. In this study, the CCME WQI was calculated based on the intended use of water and variations in physicochemical parameters and metals, following the methodology established by the CCME^14^. Various standards were used for assessing the suitability of water for drinking^16,17^, irrigation^18^, and aquatic life^14^ in the two studied habitats.

### Quantitative Real-Time PCR

Liver samples from *C. gariepinus* (*n* = 3) were collected quarterly (on the 27th of the third month) from each habitat and homogenized individually in 700 µL of Buffer TR (Vivantis Technologies, Malaysia) for total RNA extraction. One microgram of RNA was reverse transcribed using the FastQuant RT kit (TIANGEN Biotech, China). qRT-PCR was conducted using the Maxima SYBR Green qPCR Master Mix (2X) kit (Thermo Scientific), cDNA, and primers targeting genes associated with oxidative stress (*sod1*, *gpx1b*,* catalase*), metal toxicity (*mt*), inflammation (*il1b*, *nf-κb2*, *il-8*, *tnf-α*), heat shock response (*hsp70*), and xenobiotic metabolism (*cyp1a*), as well as a reference gene (*β-actin*; Table 1). The qRT-PCR thermal cycling protocol included an initial denaturation at 95 °C for 10 min, followed by 40 cycles of denaturation (15 s at 95 °C), annealing (30 s at 58 °C), and extension (30 s at 60 °C). Each sample was analyzed in triplicate, and a melting curve analysis was conducted post-reaction to verify the specificity and accuracy of the gene products. Gene expression levels were normalized to *β-actin*, and relative quantification was calculated using the 2-ΔΔCT method^19^.

**Table 1:** Primer sequences, amplicon sizes, GenBank accession numbers, and references used for the studied genes.

Gene	Forward Primer	Reverse Primer	Size	GenBank	Reference
*β-actin*	GCGTGACATCAAGGAGAAG	CAAGACTCCATACCCAAGAAAG	189	HM768299.1	^73^
*mt*	ACTGCCAGTGCAAATCCTG	TCCTGAGGCACACTTACTGC	74	KU999947.1	^74^
*catalase*	CTGGGACCTGACAGGCAATA	CTCCAGAAGTCCCACACCAT	135	MW752164.1	^42^
*cyp1a*	CCAGCACGAGCATGAAGAAA	ATGCTCTTTGACCAGCCTCT	77	MH760777.1	^42^
*sod1*	TGCTCCCGTAGTGGTTAAAGGG	TTCATCAAGTGGCCCACCATG	154	MK112879.1	^75^
*gpx1b*	ACCTGACCGCTGACATAGAG	ACATCAGACAGCCCTTCACA	180	NM_001200741.1	^42^
*il1b*	TGCAGTGAATCCAAGAGCTACAGC	CCACCTTTCAGAGTGAATGCCAGC	128	MH341527.1	^75^
*nf-κb2*	TACAGGACGAGAACGGAGACACG	TGGCTGAGGTGGTTGAACTTGTTG	136	XM_053501655.1	^76^
*il-8*	TCGCCGTATCGGGAAAATGA	GGTGCTTTAGGGTCCAGACA	136	XM_053480225.1	^76^
*hsp70*	ATGAACCCCACCAACACAAT	ATGACCTTGAAAGGCCAATG	104	DQ885945.1	^77^
*tnf-α*	TCTCAGGTCAATACAACCCGC	GAGGCCTTTGCGGAAAATCTTG	125	KM593875.1	^78^

### Identification of ectoparasites and Digenean metacercariae

To collect the ectoparasites and digenean metacercariae, individual fish were placed in a dissecting dish, their heads were severed with a sharp knife, and the gills and fins were excised with fine scissors while the skin was scraped with a sharp blade. The gills, fins, and skin scrapings were individually placed in Petri dishes with filtered water and examined for parasites using a fine needle and a stereomicroscope. The parasites were then examined under a phase-contrast microscope, and the number of parasites on each gill was recorded. Identification of *Quadriacanthus* species followed the criteria of Paperna^20^ and El-Naggar and Serag^21^, while *Gyrodactylus* sp. was identified according to Ergens^22^. Identification of *Macrogyrodactylus congolensis* and *M. clarii* was based on Gussev^23^, while the metacercaria of *Centrocestus formosanus* was identified according to Yousif et al.^24^. Ectoparasites and digenean metacercariae were counted for each individual fish, and prevalence (%), mean intensity, and abundance were calculated following Bush et al.^25^. Prevalence indicates the percentage of infected fish, while mean intensity reflects the average number of parasites per infected fish. Abundance refers to the mean parasites per fish examined, including uninfected ones.

### Statistical analysis

For non-parasitological analyses, all samples were analyzed in triplicate, with data expressed as mean ± standard deviation (Mean ± SD). Statistical analysis was performed using SPSS Software (version 25) to evaluate differences between the two sites in physicochemical parameters, heavy metal concentrations, and relative gene expression levels. Student’s *t*-test was applied, with probability values of ≤ 0.05 deemed significant and those > 0.05 considered non-significant.

For parasitological analyses, Quantitative Parasitology (QPweb; https://www2.univet.hu/qpweb/qp10/index.php) was used for analyzing the parasite data^26^. Instead of standard deviation, confidence intervals (Wald method) were reported for prevalence, and bootstrap bias-corrected and accelerated (BCa) 95% confidence limits were reported for mean intensity and abundance. For statistical analysis, a chi-square test was used to compare prevalence, while bootstrap two-sample t-tests (each with 2,000 replicates) were performed to compare mean intensity and abundance, with probability values of ≤ 0.05 considered significant and those > 0.05 deemed non-significant.

## Results

A total of 198 specimens of the clariid catfish *C. gariepinus* Burchell, 1822 were collected from Autumn 2021 to Summer 2022 from two habitats across two governorates: Dakahlia Governorate, represented by the River Nile, and Kafr El Sheikh Governorate, where the Kitchener Drain (downstream sector) joins the Mediterranean coast at Baltim.

### Physicochemical parameters of investigated habitats

Table 2 shows the physical environmental factors of water from the River Nile and the downstream stretch of the Kitchener Drain. The drain showed significantly higher turbidity and lower depth compared to the Nile. Water temperature and pH levels were relatively similar across both habitats. However, EC was significantly higher in the Kitchener Drain, signifying greater ionic content, whereas the Nile recorded significantly higher levels of DO.

**Table 2:** Physicochemical water parameters. * indicates significant (*p* ≤.05) differences compared with the Nile.

Parameter	River Nile	Kitchener
Turbidity (cm)	127.50 ± 9.57	32.50 ± 5.00^*^
Depth (m)	2.94 ± 0.43	2.05 ± 0.61^*^
Temp. (°C)	27.18 ± 5.78	25.68 ± 6.12
pH	8.60 ± 0.29	8.71 ± 0.36
EC (dS/m)	0.33 ± 0.09	2.72 ± 1.49^*^
DO (mg/L)	7.25 ± 1.32	5.65 ± 1.06^*^

Table 3 shows the water’s chemical environmental parameters of the River Nile and the drain. Generally, the Kitchener Drain had much higher concentrations of the chemical contents than the River Nile. Among them, there were significantly higher values for the total dissolved solids (TDS), the chlorides, the sulfates, and the bicarbonates. Concentrations of both monovalent cations (e.g., sodium and potassium) and divalent cations (e.g., calcium and magnesium) were significantly elevated in the Kitchener Drain. Additionally, nitrogen and phosphorus were also significantly higher in the Kitchener Drain.

**Table 3:** Concentrations of major ions and nutrients. * indicates significant (*p* ≤.05) differences compared with the Nile.

Parameter (ppm)	River Nile	Kitchener
TDS	212.33 ± 62.13	1739.53 ± 953.11^*^
HCO₃	24.78 ± 11.87	87.43 ± 46.77^*^
Cl	157.56 ± 56.18	1233.57 ± 743.00^*^
SO₄	30.00 ± 14.92	418.51 ± 164.11^*^
Ca	13.88 ± 3.79	73.97 ± 37.11^*^
Mg	9.05 ± 3.44	57.39 ± 37.48^*^
Na	183.64 ± 56.08	1597.55 ± 880.03^*^
K	5.75 ± 0.65	10.63 ± 1.65^*^
N	3.94 ± 1.40	11.66 ± 5.94^*^
P	0.46 ± 0.31	0.83 ± 0.37^*^

### Heavy metals

The subsurface water of the Kitchener Drain tended to have a higher level of many heavy metals compared with the River Nile. Aluminum, vanadium, silver, chromium, iron, copper, lead, and strontium all appeared in significantly larger quantities in the Kitchener Drain (Table 4). Selenium, cadmium, mercury, barium, gallium, manganese, zinc, bismuth, and arsenic had non-significant differences in both ecosystems, with a degree of variation. Cobalt and nickel appeared in significantly larger quantities in the River Nile. Some metals, such as boron, indium, and lithium, did not appear in either place.

**Table 4:** Heavy metals concentrations. * indicates significant (*p* ≤.05) differences compared with the Nile.

Parameter (ppm)	River Nile	Kitchener
Al	105.430 ± 102.619	279.710 ± 261.537^*^
Se	7.625 ± 6.543	10.062 ± 6.567
V	1.730 ± 0.516	3.327 ± 1.619^*^
Hg	0.042 ± 0.084	0.008 ± 0.015
Ag	0.213 ± 0.425	0.348 ± 0.406^*^
B	ND	ND
Ba	0.037 ± 0.045	0.043 ± 0.085
Cd	0.093 ± 0.086	0.082 ± 0.079
Co	0.081 ± 0.063	0.056 ± 0.065^*^
Cr	0.529 ± 0.449	0.878 ± 0.255^*^
Cu	0.205 ± 0.295	0.432 ± 0.317^*^
Fe	6.508 ± 1.003	9.923 ± 3.578^*^
Ga	3.766 ± 3.706	5.489 ± 5.534
In	ND	ND
Li	ND	ND
Mn	0.280 ± 0.095	0.329 ± 0.188
Ni	0.156 ± 0.313	ND
Pb	0.517 ± 0.340	1.024 ± 0.530^*^
Sr	0.447 ± 0.094	0.920 ± 0.278^*^
Zn	3.996 ± 0.847	4.348 ± 1.383
As	0.353 ± 0.326	0.210 ± 0.420
Bi	4.055 ± 2.003	4.524 ± 3.639

### Water quality index

**Fig. 2:**
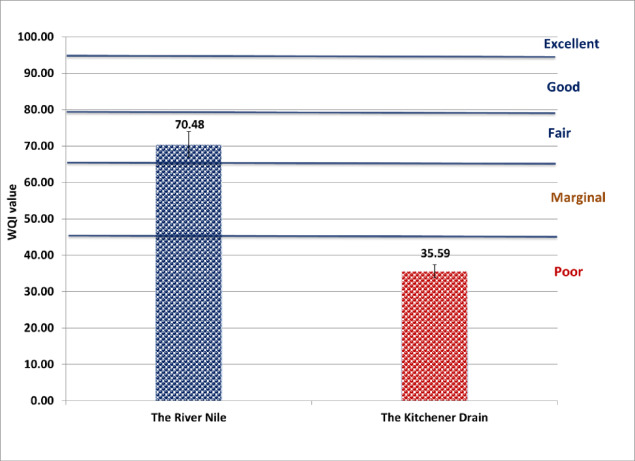
Water quality index for the River Nile and Kitchener Drain based on Egyptian drinking water guidelines^16^.

**Fig. 3:**
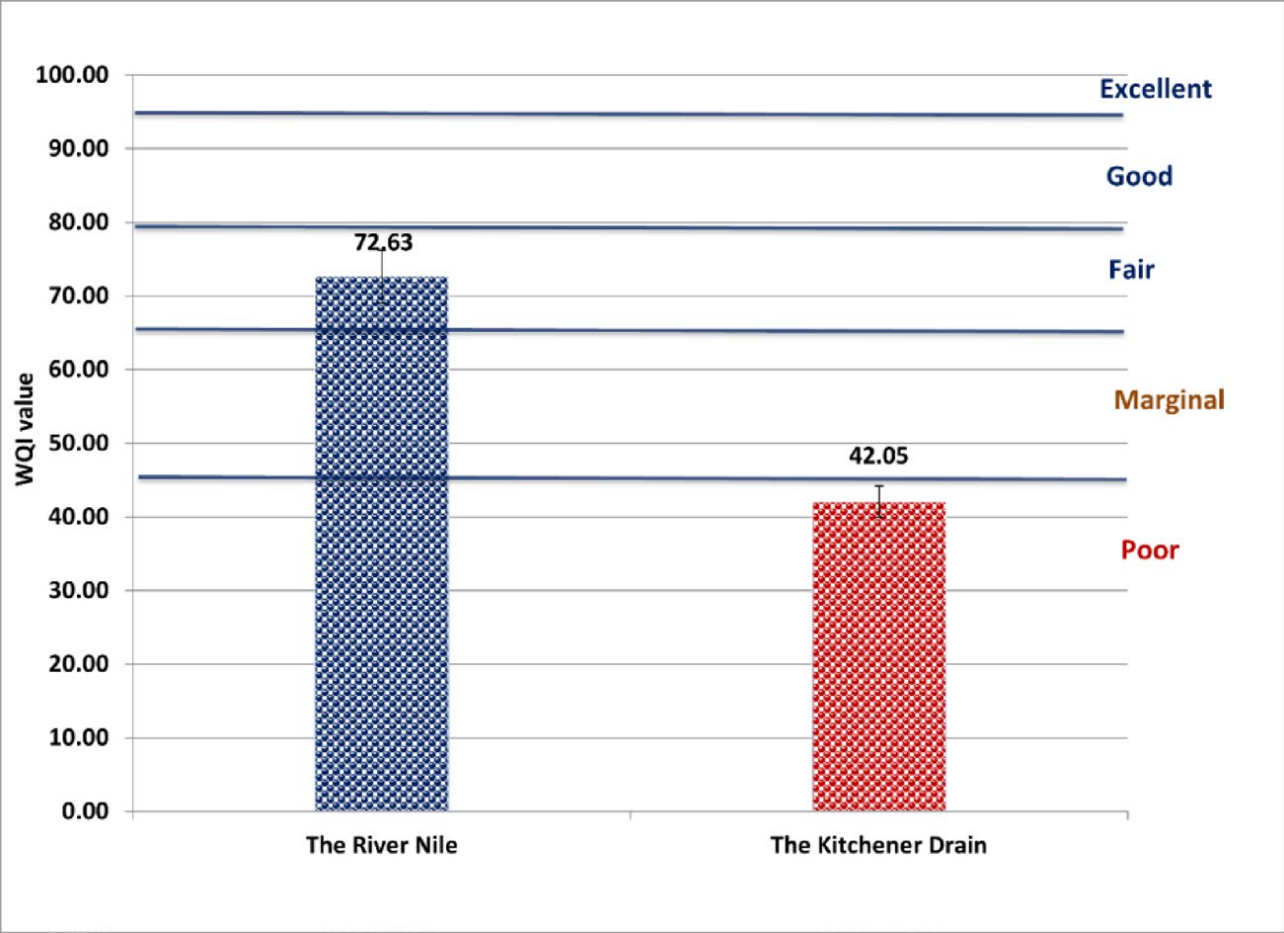
Water quality index for the River Nile and Kitchener Drain based on drinking water guidelines established by World Health Organization^17^.

**Fig. 4:**
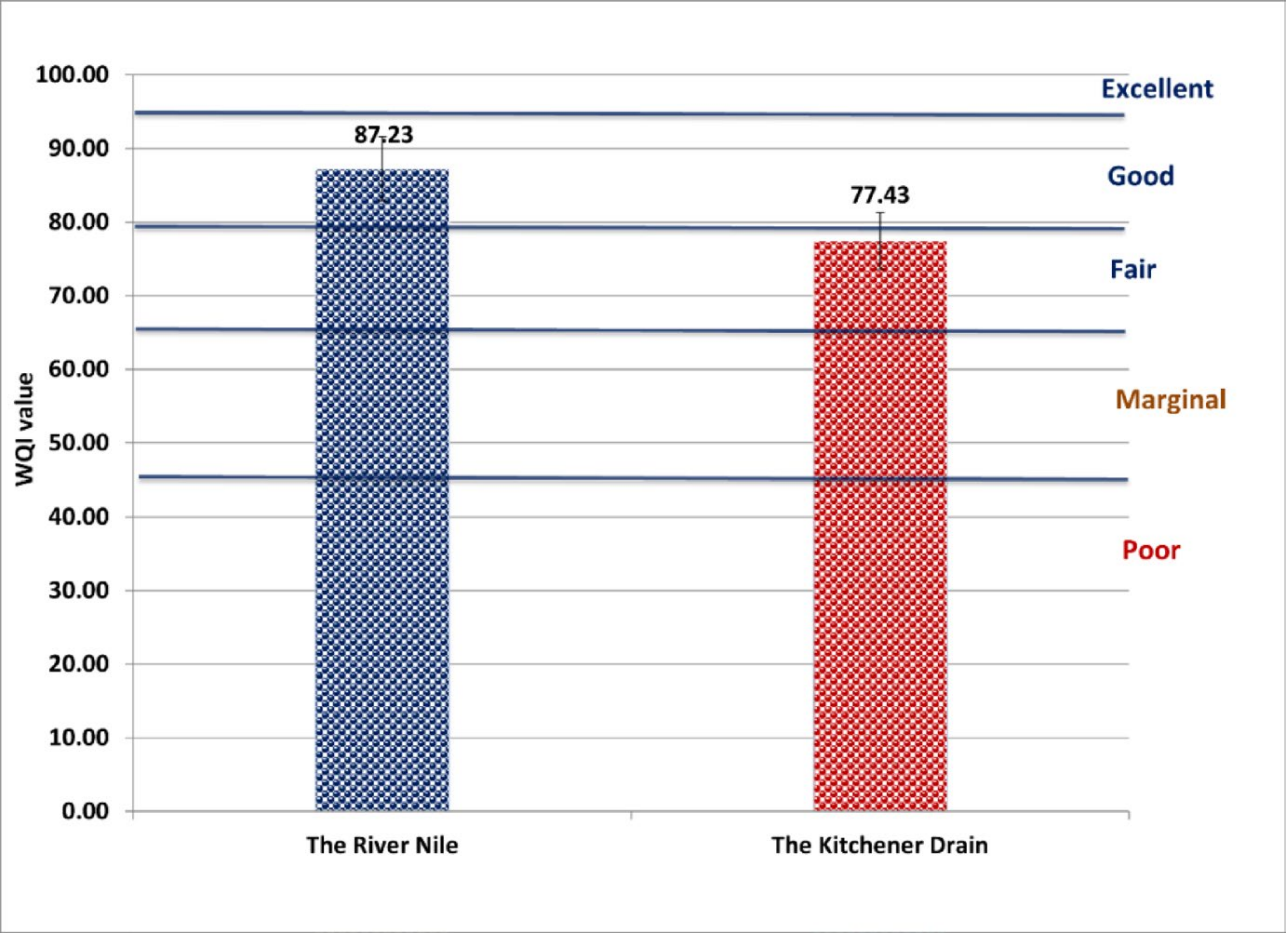
Water quality index for the River Nile and Kitchener Drain based on irrigation water guidelines established by Food and Agriculture Organization^18^.

**Fig. 5:**
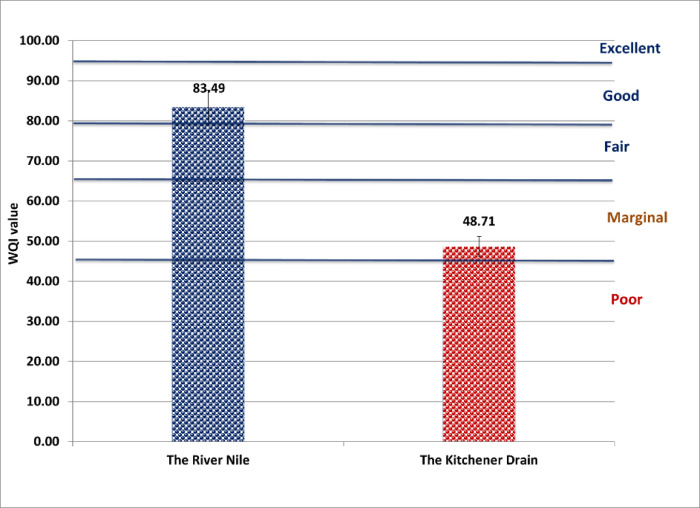
Water quality index for the River Nile and Kitchener Drain based on aquatic life criteria established by Canadian Council of Ministers of the Environment^14^.

According to drinking water guidelines provided by both the Egyptian Drinking Water Quality Standards (Fig. 2) and the World Health Organization (Fig. 3), the River Nile was classified as “fair,” while the Kitchener Drain was classified as “poor.” Under the Food and Agriculture Organization irrigation guidelines, the River Nile was categorized as “good,” whereas the Kitchener Drain was rated as “fair” (Fig. 4). In terms of aquatic life protection, the River Nile met the criteria for “good” water quality, whereas the Kitchener Drain was classified as “marginal” (Fig. 5).

### Quantitative Real-Time PCR

*C. gariepinus* from the Kitchener Drain exhibited significantly higher hepatic expressions of *sod1*, *gpx1b*, *catalase*, *mt*, *il1b*, *nf-κb2*, *il-8*, *tnf-α*, *hsp70*, and *cyp1a*. *mt* had the highest relative expression. *cyp1a* showed the least relative expression (Table 5).

**Table 5:** Relative expressions of sod1, gpx1b, catalase, mt, il1b, nf-κb2, il-8, tnf-α, hsp70, and cyp1a. * indicates significant (*p* ≤.05) differences compared with the Nile.

	Relative expression
*mt*	5.42 ± 3.14^*^
*gpx1b*	2.96 ± 1.17^*^
*hsp70*	2.67 ± 1.31^*^
*il-8*	4.08 ± 3.78^*^
*tnf-α*	2.66 ± 0.54^*^
*sod1*	3.43 ± 1.85^*^
*il1b*	4.82 ± 3.97^*^
*cyp1a*	1.73 ± 0.72^*^
*nf-κb2*	3.78 ± 2.49^*^
*catalase*	3.31 ± 0.91^*^

### Total prevalence (%), mean Intensity, and abundance values of monogeneans and digenean metacercariae in *Clarias Gariepinus*

**Fig. 6:**
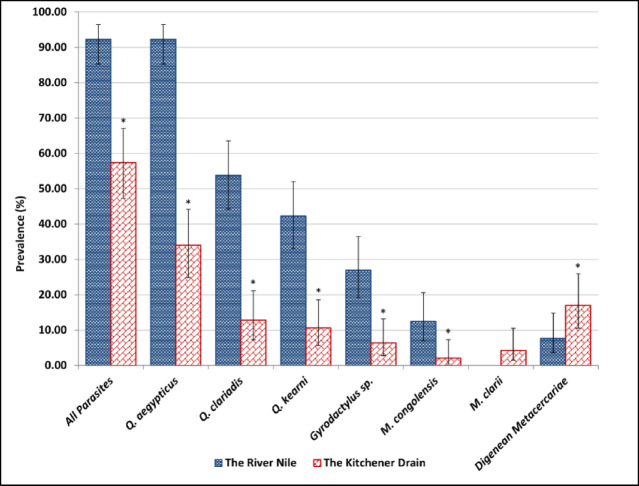
Mean prevalence of Quadriacanthus aegypticus, Q. clariadis, Q. kearni, Gyrodactylus sp., Macrogyrodactylus congolensis, M. clarii, and digenean metacercariae infecting Clarias gariepinus collected from the River Nile and the Kitchener Drain; * indicates significant (*p* ≤.05) differences compared with the Nile per chi-square test.

Ectoparasites belonging to monogenean species and digenean metacercariae were recorded in *C. gariepinus* from both the river and the drain. Ectoparasites in *C. gariepinus* were significantly more prevalent in specimens from the Nile compared to those from the drain (Fig. 6). Among the detected species, *Quadriacanthus aegypticus* showed the highest prevalence in the River Nile, while it was moderately present in the Kitchener Drain. *Macrogyrodactylus congolensis* was rare in both habitats, and *M. clarii* was not detected in the drain. The Kitchener Drain had a significantly higher prevalence of metacercariae of the endoparasite *Centrocestus formosanus* compared to the Nile River, although their overall prevalence remained low.

**Fig. 7:**
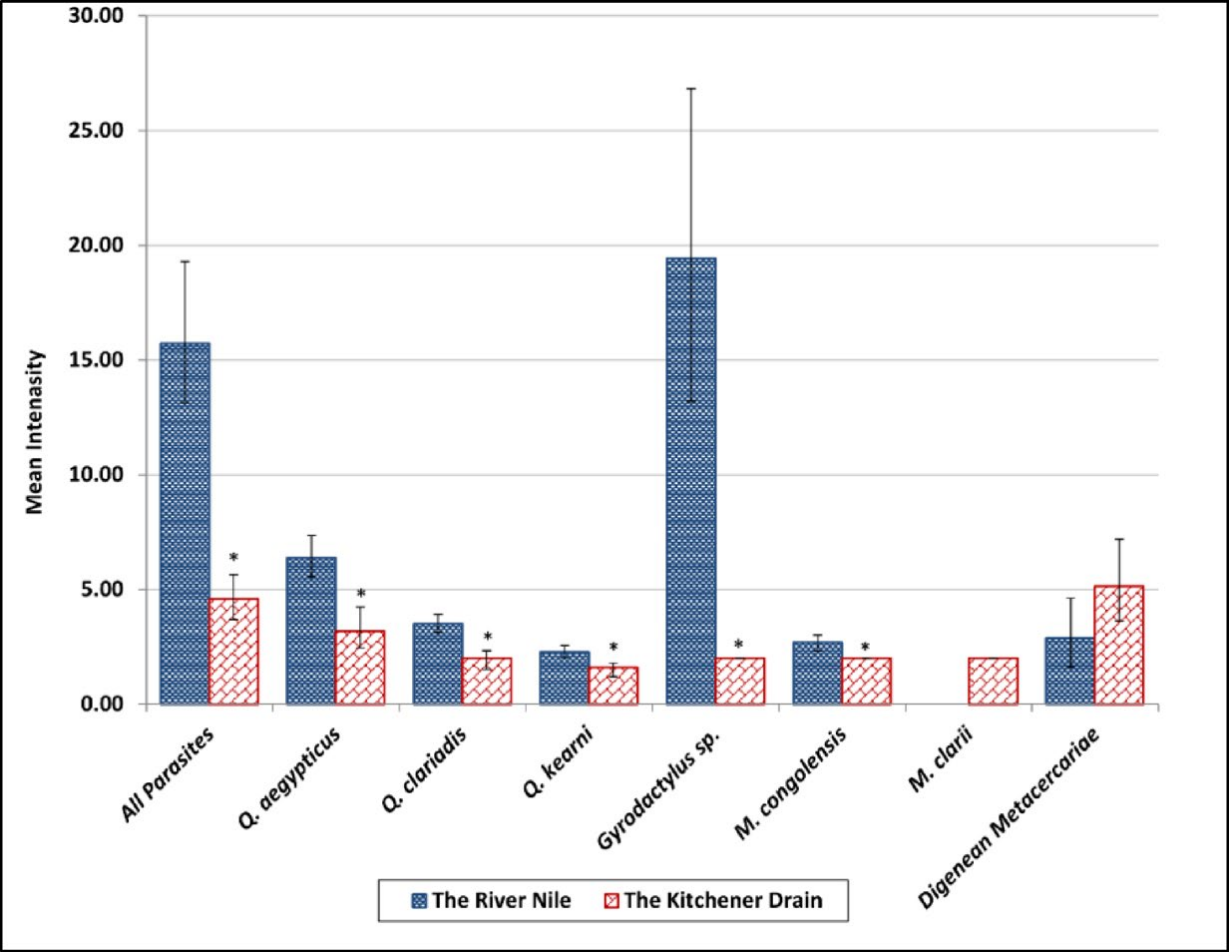
Mean intensity of Quadriacanthus aegypticus, Q. clariadis, Q. kearni, Gyrodactylus sp., Macrogyrodactylus congolensis, M. clarii, and digenean metacercariae infecting Clarias gariepinus collected from the River Nile and the Kitchener Drain; * indicates significant (*p* ≤.05) differences compared with the Nile per bootstrap 2 sample *t* test.

The mean intensity of ectoparasite infestation was significantly greater in the River Nile than in the drain. *Q. aegypticus* and *Gyrodactylus* sp. exhibited the highest mean intensity in the Nile (Fig. 7). *Q. kearni* showed moderately elevated intensity in the River Nile. In the Kitchener Drain, intensity values remained consistently lower across most species. *M. congolensis* displayed an unusually high mean intensity in a single case from the Kitchener Drain but was otherwise limited in occurrence. *M. clarii* had a very low mean intensity overall, and it was not detected in the River Nile. The drain had a higher intensity of metacercariae of the endoparasite *C. formosanus* compared to the Nile River, but the difference was not statistically significant.

**Fig. 8:**
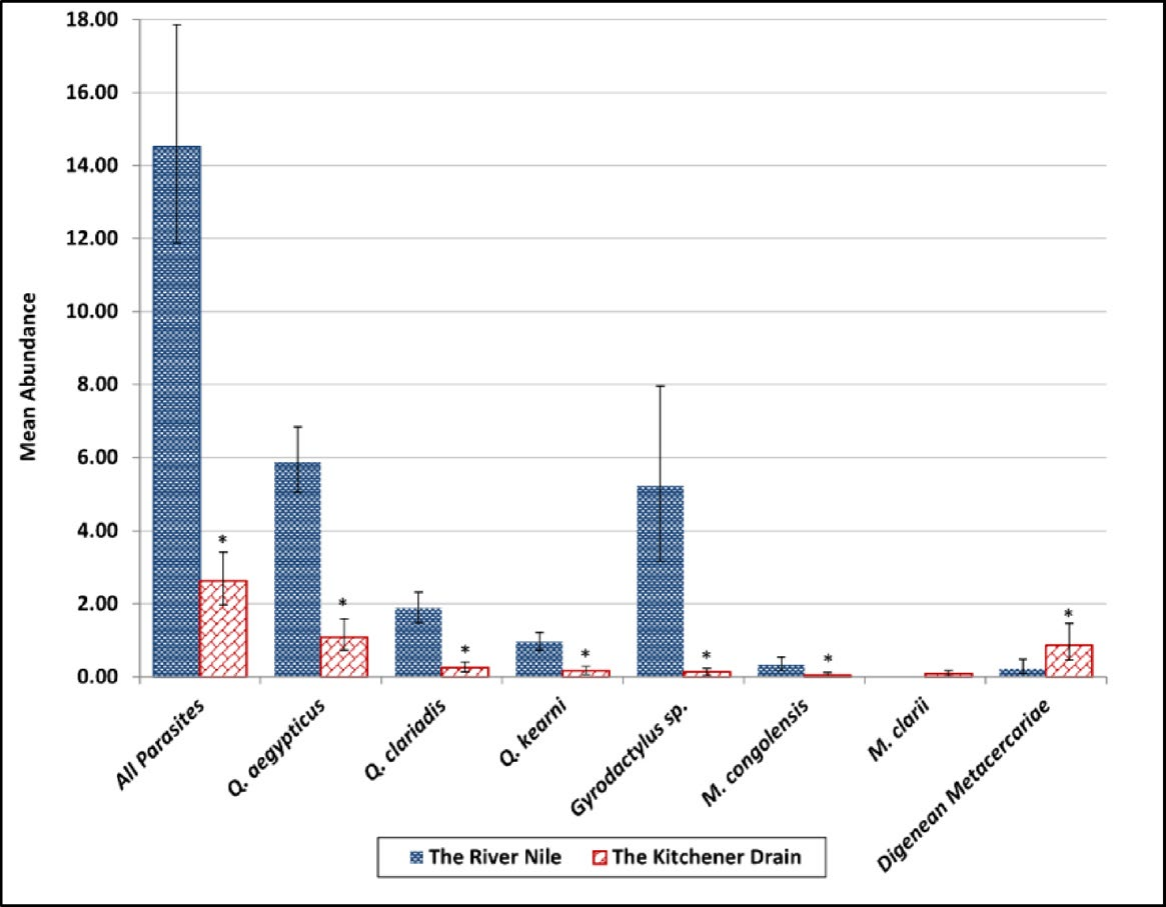
Mean abundance of Quadriacanthus aegypticus, Q. clariadis, Q. kearni, Gyrodactylus sp., Macrogyrodactylus congolensis, M. clarii, and digenean metacercariae infecting Clarias gariepinus collected from the River Nile and the Kitchener Drain; * indicates significant (*p* ≤.05) differences compared with the Nile per bootstrap 2 sample *t* test.

Abundance values followed the same trend as prevalence and intensity, with significantly higher values recorded in the River Nile (Fig. 8). *Q. aegypticus* was the most abundant species in both habitats. *Q. kearni* showed modest abundance in both habitats. *M. congolensis* and *M. clarii* were both of low abundance overall, but *M. clarii* was entirely absent from the River Nile. The drain had a significantly higher abundance of metacercaria of the endoparasite *C. formosanus* compared to the Nile River.

## Discussion

The physical characteristics of the River Nile and Kitchener Drain were evaluated by measuring depth, turbidity, pH, water temperature, EC, and DO. Evidence indicates that the Kitchener Drain has increased turbidity and EC compared with the River Nile due to the possible accumulation of increased sediment and salt deposits. This can be linked with runoff coming from agriculture and industry^1^. Shallowness of the drainage and the organic load further increase oxygen depletion since the decomposition by microbes uses most of the oxygen present^27^. The relative similarity in the pH and water temperature between the two habitats is regulated by the general environment of the area rather than site-specific factors. Nevertheless, the increased DO of the River Nile indicates a healthier aquatic environment in comparison with the drain. These results are in agreement with recent Egyptian studies, indicating that drains that receive agricultural and sewage effluents tend to have increased suspended solids and salinity, as well as low DO rates^1,28^.

The measured chemical parameters were TDS, bicarbonates, chlorides, sulfates, calcium, magnesium, sodium, potassium, nitrogen, and phosphorus. The Kitchener Drain contained significantly higher concentrations of the majority of the parameters compared to the River Nile. Abdelhafiz et al.^28^ linked high TDS and ionic composition in such water bodies with agricultural runoff as well as inadequate management of wastes. Elevated nitrogen and phosphorus concentrations in the drain, as postulated by Qi et al.^29^, may be attributed to agricultural runoff with fertilizers. Fertilizer-rich wastewater accelerates the rate of eutrophication^30^, as indicated by the low DO concentration in the drain. The River Nile contained low concentrations of the above parameters, indicating better water quality.

The subsurface water of the Kitchener Drain showed significantly higher heavy metal concentrations than the Nile. This likely reflects heavy pollution inputs from untreated sewage, industrial effluents, and agricultural runoff in the drain. This agrees with past findings that Delta drains (including Kitchener) accumulate high levels of metals such as Cd, Pb, Zn, and Cr near pollution sources​^31–33^. The Nile tended to dilute contaminants overall, although elements such as Co and Ni showed a relative abundance in the river water, likely due to distinct upstream sources or enhanced water-column mobility. In the same way, heavy metal monitoring in the Nile recorded peak metal values in locations with sewage, industrial wastes, and farm drainages, illustrating the capacity of drain discharges to increase metal burdens in the river^34,35^. Some metals (e.g., Se, Cd, Ba, Mn, and As) had the same order of magnitude of concentrations in both habitats and therefore suggest broad regional sources (e.g., phosphate fertilizers)^36^. Metal pollution is environmentally serious because toxic metals can accumulate in soil, crops, and aquatic food chains and pose a threat to the health of ecosystems and human well-being.

The CCME WQI results for the Nile and the drain agree with the trend presented in recent Egyptian water quality studies. A “fair” WQI for the Nile means it marginally meets guidelines and often requires treatment before it is safe for human consumption. This is comparable to other WQI studies of the Nile River^37^, which typically grade the main river from “fair” to “good” for household consumption. In contrast, the drain’s “poor” WQI for drinking indicates it fails to meet basic safety thresholds, reflecting contamination by untreated sewage and industrial effluents​^27^. Ameen et al.^27^ similarly reported a very low WQI in Kitchener’s water, underscoring that the drain water is largely unfit without extensive treatment.

For irrigation purposes, water quality is judged by more lenient criteria. Accordingly, the Damietta branch was rated “good” under the Food and Agriculture Organization^18^ irrigation standards, indicating it can be used for crops with minimal risk of salinity or toxicity issues. The polluted Kitchener water marked “fair” for irrigation, reflecting that many chemical parameters fall within allowable ranges for agricultural use. However, long-term use of such marginal-quality drain water poses concerns for soil health and crop uptake of pollutants^38^. These findings echo national water-reuse studies that caution against using untreated drainage water for irrigation without dilution or treatment measures^39^.

For aquatic life protection, the CCME WQI results signal better conditions in the Damietta branch than in the drain. The Damietta branch’s classification as “good” for aquatic life implies that most parameters (e.g., DO, nutrients, and metals) remain within tolerable levels for ecosystem health. This agrees with observations that the Nile’s main channels can support diverse aquatic biota except near pollution point sources​^40^. In contrast, the drain was rated “marginal” for aquatic life, reflecting frequent exceedances of ecological water-quality criteria (such as chronic low DO and elevated ammonia) that stress or eliminate sensitive species. Such degraded quality is typical of heavily polluted Delta drains, where biodiversity loss and fish deaths have been documented due to high pollutant loads^38^. These results underscore the need for improved wastewater treatment and pollution control in Nile Delta drains to safeguard water use for drinking, sustainable irrigation, and aquatic ecosystem health in Egypt.

All the genes were upregulated in the fish from the drain, suggesting significant environmental stress. The antioxidant enzymes *sod1* (superoxide dismutase 1), *gpx1b* (glutathione peroxidase 1b), and *catalase* were upregulated significantly. These enzymes are generally induced in fish challenged with xenobiotics, heavy metals, ammonia, or nitrites, leading to oxidative stress^41^. Superoxide dismutase converts superoxide radicals into hydrogen peroxide, which glutathione peroxidase and catalase subsequently neutralize. Ibor et al.^42^ noted increased expression of *sod1*, *gpx1b*, and *catalase* in *C. gariepinus* exposed to simulated leachate from a solid waste dumpsite in Calabar, Nigeria. Similarly, Parida and Sahoo^41^ noted the induction of *gpx* expression in *C. gariepinus* from a contaminated rivulet near Narora, India.

Metallothionein (*mt*) was the most upregulated in the study, suggesting significant heavy metal contamination. As a metal-binding, cysteine-rich protein, metallothionein plays a critical role in detoxifying metals such as cadmium, copper, and zinc in aquatic organisms^43^. The critical upregulation of *mt* implies that heavy metal contamination is a dominant stressor in this study. Previous studies on teleost species, such as *C. gariepinus*, have evidenced *mt* induction for acute as well as chronic exposure to metals^4,42,44,45^. *cyp1a* (cytochrome P450 1 A) showed a more modest upregulation. Cytochrome P450 1 A is a key enzyme in the metabolism of organic pollutants, such as polycyclic aromatic hydrocarbons^42^. However, its lower induction in this study may indicate that organic pollutants are not the dominant stressor or that exposure to stressors for a long period reduces the expression of *cyp1a*, as reported by Quirós et al.^46^ and Salem et al.^4^. *hsp70* (heat shock protein 70) was also upregulated, suggesting a general cellular stress response. Heat shock protein 70 acts as a molecular chaperone, assisting in protein folding and cell protection against stressors such as chemical exposure, heat, or hypoxia^47,48^. Induction of *hsp70* was reported by Rangaswamy et al.^48^ in fish exposed to deteriorated water quality with excess ammonia, phosphorus, and suspended solids.

Multiple pro-inflammatory markers were significantly upregulated, including *il1b*, *nf-κb2*, *il-8*, and *tnf-α*. This elevation reflects an active inflammatory response. Interleukin-1β (*il1b*) and tumor necrosis factor-alpha (*tnf-α*) are pro-inflammatory cytokines^49,50^, interleukin-8 (*il-8*) acts as a chemokine^51^, and nuclear factor kappa B (*nf-κb2*) is a transcription factor for immune and inflammatory pathways^52^. Induced inflammation in fish was associated with exposure to heavy metals in aquatic environments in numerous studies^53–56^.

This gene expression profile reveals a complex response to environmental stress, characterized by enhanced antioxidant defense, detoxification, inflammation, and cellular protection. This indicates that *C. gariepinus* in the drain is under considerable environmental stress, likely driven by heavy metal contamination. These findings align with the high levels of metals and suspended matter detected in the drain.

Monogenean parasites are highly responsive to changes in water’s chemical and physical characteristics, as well as heavy metal levels, positioning them as valuable bioindicators for evaluating the health of ecosystems^6,57^. According to Sue et al.^58^, monogenean fish ectoparasites are impacted by aquatic pollution in two ways: directly, through effects on the parasites themselves, or indirectly, by altering the immune responses of their fish hosts. As a result, whether the negative impact on the host’s immune system outweighs the direct effects on the parasite, ectoparasite populations may either increase or decrease. Some research indicates that monogenean abundance rises with higher pollution levels, while other studies suggest that pollution can reduce their numbers.

In the present study, the River Nile (less polluted) supported a higher diversity and intensity of monogenean infestations, with species such as *Q. aegypticus*, *Q. clariadis*, and *Gyrodactylus* sp. showing significantly greater prevalence, intensity, and abundance in this habitat compared with the Kitchener Drain (more polluted). These differences may be attributed to the relatively better water quality, stable ecological conditions, and possibly denser host populations in the River Nile, which are conducive to parasite transmission and development. In contrast, the Kitchener Drain, characterized by higher levels of pollution and fluctuating water parameters, appeared to exert limiting effects on parasite establishment, particularly for species like *Q. clariadis* and *Gyrodactylus* sp., whose presence was considerably reduced. Notably, *M. clarii* was detected only in the Kitchener Drain, although at low levels, suggesting that certain parasites may tolerate or prefer degraded conditions.

Many laboratory and field studies support the findings of the present study that pollution may negatively impact the distribution of ectoparasites. For example, Sanchez-Ramirez et al.^59^ in a laboratory experiment found that the abundance of the monogenean *Ancyrocephalinae* decreased at the highest levels of hydrocarbon pollution. In a field study, Madanire-Moyo and Barson^7^ found that at the relatively unpolluted sites, *C. gariepinus* harbored a rich community of ectoparasites, including gill monogeneans such as *Dolops ranarum*, *Lamproglena clariae*, *Chonopeltis* sp., and *Macrogyrodactylus* sp. They further clarified that the distribution and occurrence of the ectoparasite *Macrogyrodactylus* sp. were limited to the unpolluted sites, demonstrating their sensitivity to organic pollution^7^. Similarly, Madanire-Moyo et al.^8^ reported that tilapia from an unpolluted dam harbored numerous gill monogeneans, while fish from a mine wastewater dam (heavy metal pollution) had lost many of these parasites. Madanire-Moyo et al.^8^ further noted that in the South African reservoirs, at least four *Cichlidogyrus* species were absent from the heavily impacted mine site but present (with high prevalence and intensity) in the reference site. In another field study, Falkenberg et al.^60^ mentioned that parasite species richness was significantly lower in the most polluted study area.

The findings of this study indicate that the metacercaria of the digenean endoparasite *C. formosanus* is significantly more prevalent in the highly polluted aquatic habitat (the Kitchener Drain). This trend aligns with previous research demonstrating that trematode parasitic infections often increase in environments degraded by anthropogenic pollution^64,65^. Pollution, particularly from organic waste and heavy metals, can alter host-parasite dynamics by compromising the immune responses of intermediate hosts, such as mollusks and fish, making them more susceptible to parasitic infections^66,67^. *C. formosanus* thrives in such conditions due to increased intermediate host vulnerability as pollutants like heavy metals (e.g., lead) and organic effluents weaken the immune defenses of snails (e.g., *Melanoides tuberculata*)^68,69^, facilitating higher cercarial production^70^ and reduced predator pressure as polluted habitats often experience declines in predatory fish that regulate snail populations, indirectly benefiting trematode transmission^71^. These mechanisms suggest that *C. formosanus* benefits from ecological disturbances caused by pollution, increasing its transmission success. This has significant implications for public health, as this parasite causes centrocestiasis in humans, primarily through the consumption of raw or undercooked infected fish^72^.

In conclusion, the study demonstrates the effectiveness of integrating molecular biomarkers and parasitic infestation to assess aquatic ecosystem health and water quality. The Kitchener Drain exhibited elevated ionic content, nutrient loading, and heavy metal concentrations and consequently showed poorer water quality than the River Nile, as reflected in its lower CCME WQI scores. *C. gariepinus* from the drain activated several defense responses in an attempt to cope with the loss of oxygen, exposure to toxic chemicals, and other forms of environmental stress. Monogenean ectoparasites were more prevalent, intense, and abundant in the Nile, reflecting their sensitivity to the degraded conditions in the drain. In contrast, metacercaria of the digenean endoparasite *C. formosanus* was more prevalent in the polluted drain, likely benefiting from ecological imbalances caused by contamination. This integrated bioassessment can, therefore, serve as an early warning system for water quality deterioration, guiding targeted management and pollution control measures. Lastly, there is an urgent need to improve wastewater treatment and reduce pollutants in drainage systems like the Kitchener Drain in order to protect aquatic biodiversity, fisheries, and consumers’ health.

## Data Availability

The data supporting the results of this study are available from the corresponding author upon reasonable request.
